# Attempted infanticide and suicide inaugurating catatonia associated with Hashimoto’s encephalopathy: a case report

**DOI:** 10.1186/s12888-016-0719-7

**Published:** 2016-01-19

**Authors:** Laurence Lalanne, Marie-Emmanuelle Meriot, Elisabeth Ruppert, Marie-Agathe Zimmermann, Jean-Marie Danion, Pierre Vidailhet

**Affiliations:** Department of Psychiatry, Fédération de Médecine Translationelle de Strasbourg (FMTS), University Hospital of Strasbourg, Strasbourg, France; INSERM 1114, Fédération de Médecine Translationelle de Strasbourg, University Hospital of Strasbourg, Strasbourg, France; Department of Neurology, Sleep and Electrophysiology Clinic, Fédération de Médecine Translationelle de Strasbourg (FMTS), University Hospital of Strasbourg, Strasbourg, France; Institute for Cellular and Integrative Neurosciences, CNRS - UPR 3212, Fédération de Médecine Translationelle de Strasbourg (FMTS), 67084 Strasbourg, France; INSERM1114-Department of Psychiatry, University of Strasbourg- 1 place de l’hôpital, 67000 Strasbourg, France

**Keywords:** Catatonia, Hashimoto’s encephalopathy, Post-partum auto-immune disease, Psychosis

## Abstract

**Background:**

Catatonia is a neuropsychiatric syndrome with motor and behavioural symptoms. Though usually occuring in patients with schizophrenia and mood disorders, this syndrome may also be associated with neurological diseases or general medical conditions. Few cases of catatonia associated with autoimmune disorders have been described.

**Case presentation:**

Here, we report the case of a 27-year-old woman diagnosed with Hashimoto’s encephalitis (HE) who attempted suicide and infanticide by defenestration. As she presented risk factors for postpartum psychosis, she was treated principally with antipsychotics. Despite adequate treatment for psychosis, symptoms worsened and she developed catatonia. Complementary investigations showed elevated titres of anti-thyroglobulin and anti-thyroperoxidase antibodies (200 and 10 times, respectively, as compared to normal levels) and electroencephalography were suggestive of encephalopathy. In the presence of an otherwise unexplained neuropsychiatric condition, HE was suspected and oral prednisolone was introduced. Psychiatric symptoms improved dramatically within 72 h and the patient was still free of any symptom 3 years later.

**Conclusion:**

Catatonia of organic aetiology should always be considered before a psychiatric aetiology especially in case of clinical worsening in spite of adequate psychotropic treatment. To our knowledge, this is the first description of catatonia associated with HE.

## Background

Catatonia is a neuropsychiatric syndrome with motor and behavioural symptoms occurring in approximately 8 % of patients admitted for psychiatric illness such as schizophrenia or mood disorders [1]. Seven to 45 % of catatonias are “organic catatonias” that is, catatonia secondary to a general medical condition [1–4]. Among them, some cases of catatonia associated with autoimmune diseases have been described in systemic lupus erythematosus, anti-NMDAR (N-methyl-d-aspartate receptor) encephalitis [5–8], paraneoplasic syndrome [7, 9, 10], PANDAS (Pediatric Autoimmune Neuropsychiatric Disorder) [11] and in other rare syndromes like Wilson disease [12]. First line recommended treatementr in catatonia are benzodiazepine and ECT [13] but, in case of SLE and anti-NMDAR encephalitis in adults, etiological treatment, respectively plasma exchange and immunoglobulin perfusion associated with corticosteroid, is an efficient option avoiding using ECT and psychotropic drugs [14, 15]. Here we present a case highlighting that, as psychiatric symptoms can be prominent in encephalopathy, the presence of catatonia associated with psychotic features can be a misleading presentation for an underlying organic aetiology.

## Case presentation

In 2009, a 27-year-old woman was admitted to our psychiatric department. She had been found by emergency response firemen, sitting with her 8-month-old twin babies on the window ledge of her 9^th^ floor apartment tossing personal effects into the air.

The patient had no prior personal or family psychiatric history. She was of Algerian origin and had arrived in France 18 months earlier. Her husband was unemployed, drug-dependent, and was rarely present at home. It had been her first pregnancy, and she had given birth to two girls via caesarean section, due to uterine haemorrhage during the 35^th^ week of amenorrhoea.

Upon admission, an examination found fluctuating psychomotor agitation, anxiety, emotional lability, and impulsivity. The patient had incoherent speech, persecutory delusions, auditory hallucinations, and was disoriented in time and space. According to her family, symptoms had appeared three months earlier and had progressively worsened. She was not known to be taking any medications nor did she suffer from substance abuse. Considering the clinical picture and because of the presence of classical risk factors (first-time mother with multiple births associated with obstetrical complications, recent immigration, father of the children barely present), post-partum psychosis was firstly suspected even though the delayed onset of psychiatric symptoms 5 to 8 months after birth was longer than what is usually admitted for such a diagnosis [16]. As a consequence, oral risperidone 2 mg daily and cyamemazine 30 mg daily were introduced. Forty-eight hours later, symptoms worsened, and the patient presented with permanent auditory hallucinations, psychomotor instability, as well as aggressive behaviour alternating with mutism, negativism, stereotypic gestures and diffuse rigidity with catalepsy. A vesperal aggravation was noted. According to DSM-IV [17] and DSM-5 [18] criteria, catatonia was diagnosed. Oral lorazepam was introduced and progressively increased until 12.5 mg daily. Because the clinical picture worsened and to avoid an evolution towards malignant catatonia, antipsychotics were stopped and complementary investigations were conducted.

Electroencephalography (EEG) showed a widespread slowing of background activity without sharp-waves, suggestive of encephalopathy. Brain imaging including CT-scan, magnetic resonance imaging (MRI) scan, and single photon emission computed tomography (SPECT-scan) were all normal. The patient was euthyroid but had elevated titres of anti-thyroglobulin (TGAB) and anti-thyroperoxidase (TPOAB) antibodies (200 and 10 times compared to normal, respectively). Cerebrospinal fluid (CSF) examination revealed normal cell count, protein, and glucose levels. CSF cultures were negative and TGAB and TPOAB titres in the CSF were normal. Thyroid echography showed a heterogeneous gland without any sign of inflammation. No other abnormality was found from further testing, including blood tests for regular auto-immune disorders, serological tests for herpes simplex virus, cytomegalovirus, viral hepatitis B and C, human immunodeficiency virus, syphilis, as well as blood sampling for vitamins B 12, PP, and ceruloplasmin.

Hashimoto’s encephalopathy (HE) was suspected given the association of high concentrations of anti-thyroid antibodies and the presence of an otherwise unexplained neuropsychiatric condition. Although medication with risperidone 2 mg daily, cyamemazine 30 mg daily, and eventually lorazepam 12.5 mg daily, was ongoing, no clinical amelioration was observed. Therefore, oral prednisolone was introduced at 60 mg daily. It was quickly followed by a dramatic improvement of the clinical picture; within 72 h, the patient was again oriented in time and space, and auditory hallucinations, as well as catatonia, disappeared. A follow-up EEG returned normal. Corticosteroid treatment was maintained at a dosage of 60 mg daily for a month before being progressively decreased and stopped within a year. In 2013, after three years of follow up, the patient still remained free of any psychiatric symptom.

## Discussion

Here, we describe a case of attempted infanticide and suicide at the onset of catatonia. The literature report increased risk of suicide and infanticide in catatonia especially of psychiatric origin [19, 20]. Due to the predominance of psychiatric symptoms and the presence of multiple risk factors, our initial diagnosis was post-partum psychosis. However, treatment resistance made us suspect a catatonia of organic origin. In catatonia, a causality assessment score (CAUS) has been proposed by Consoli et al. (2012) [21]. This score takes into account the presence of similar cases described in the literature, the presence of clinical, biological and paraclinical symptoms, and the response of the patient to the specific treatment related to the suspected medical condition. When applied to our clinical case, the score is 7/10, clearly favoring an organic origin. HE is very probable in our patient given high titers of anti-thyroid antibodies and the rapid normalization of her clinical symptoms and EEG once prednisolone was introduced [22–27]. It must be noted that neither abnormal brain imaging nor the presence of anti-thyroid antibodies in the CSF are necessarily required for HE diagnosis [25]. Additionally, the thyroid status may vary from normal to pathological among patients with HE [23–25].

Hashimotos’encephalopathy is a rare syndrome, first described by Brain et al. in 1966 [28]. It is suspected whenever symptoms of acute or subacute encephalopathy are associated with high serum levels of anti-thyroid antibodies [18–21]. Because it lacks specific markers and is a clinically heterogeneous syndrome, HE remains an elusive nosologic entity [18–21]. Clinical manifestations include confusion, coma, stroke-like episodes, seizures, psychosis, dementia, myoclonus and myelopathy [22–26]. The pathophysiology of HE remains poorly understood: proposed mechanisms include cerebral vasculitis and neuronal reaction mediated by antibodies [29]. Recent publications suggested that an insufficiency of cerebral blood flow, especially the in left prefrontal cortex and anterior cingulate areas, due to vasculitis causes the neuropsychiatric symptoms in HE, like psychosis, consciousness disturbance [30] and mood disorders [31]. Besides, the litterature showed a link between depression and antithyroid antibodies regardless of thyroid status, even after adjustment of psycho-social determinants of depression [32]. However, it is still not known whether antibodies in HE may play a direct role in brain dysfunction especially as the level of circulating autoantibodies is not correlated with the severity of clinical manifestations or with response to treatment. Moreover, antithyroid antibodies can be elevated in a significant percentage of the normal population and in other thyroid disorders [25]. Laske et al (2005) reported a case of severe depression associated with HE [26] and Babtain (2010) reported a case of HE as late onset depression with psychotic features [33]. Both interpretated psychiatric symptoms as resulting from encephalopathy associated with autoimmune thyroid disease. Both of cases rapidly and dramatically improved under prednisolone, just as was seen with our clincial case. Favorable response to corticosteroids and association with other autoimmune diseases in HE point to inflammatory or autoimmune dysfunction [26, 29, 32–34] that may also underlie the pathophysiology of associated psychiatric symptoms such as depression, psychosis and catatonia. It is consistent with the current hypothesis of autoimmune and inflammatory pathophysiology of mood and psychotic disorders [34, 35].

## Conclusion

As psychiatric symptoms can be prominent in encephalopathy, they could potentially be misleading and point to catatonia of psychiatric origin. However, an organic aetiology should be always considered, especially in the absence of any mental disorder history. Hashimoto’s encephalopathy is a difficult diagnosis, especially when the thyroid hormonal status is normal. Clinicians should therefore think about dosing thyroid antibodies when confronted with catatonia. More generally, a multidisciplinary diagnosis approach with psychiatrists, internists and neurologists is recommended in any case of catatonia [36].

### Consent section

Written informed consent was obtained from the patients for publication of their case reports and any accompanying images. A copy of the written consent is available for review by the Editor of this journal.
